# The emerging potentials of lncRNA DRAIC in human cancers

**DOI:** 10.3389/fonc.2022.867670

**Published:** 2022-08-04

**Authors:** Qinfan Yao, Xiuyuan Zhang, Dajin Chen

**Affiliations:** ^1^ Kidney Disease Center, the First Affiliated Hospital, College of Medicine, Zhejiang University, Hangzhou, China; ^2^ Key Laboratory of Kidney Disease Prevention and Control Technology, Hangzhou, China; ^3^ National Key Clinical Department of Kidney Diseases, Institute of Nephrology, Zhejiang University, Hangzhou, China; ^4^ Zhejiang Clinical Research Center of Kidney and Urinary System Disease, Hangzhou, China

**Keywords:** DRAIC, lncRNA, biological function, mechanism, application

## Abstract

Long non-coding RNA (lncRNA) is a subtype of noncoding RNA that has more than 200 nucleotides. Numerous studies have confirmed that lncRNA is relevant during multiple biological processes through the regulation of various genes, thus affecting disease progression. The lncRNA DRAIC, a newly discovered lncRNA, has been found to be abnormally expressed in a variety of diseases, particularly cancer. Indeed, the dysregulation of DRAIC expression is closely related to clinicopathological features. It was also reported that DRAIC is key to biological functions such as cell proliferation, autophagy, migration, and invasion. Furthermore, DRAIC is of great clinical significance in human disease. In this review, we discuss the expression signature, clinical characteristics, biological functions, relevant mechanisms, and potential clinical applications of DRAIC in several human diseases.

## Introduction

Long non-coding RNA (lncRNA) is a type of non-protein-coding RNA that is longer than 200 nucleotides (1–5). With the advancement of genomics technology during the past few decades, several lncRNAs have become the focus of clinical research and were discovered to be closely associated with the progression of human diseases (5–8). There is growing evidence that lncRNA can actively participate in the regulation of a variety of biological functions mainly through the modification of gene expression levels (9–13). These functions include cell proliferation, apoptosis, autophagy, metabolism, invasion, and migration.

The lncRNA DRAIC (Downregulated RNA In Cancer) is a 1.7 kb lncRNA located on the human chromosome 15q23 (14). lncRNA DRAIC was first discovered to act as a tumor suppressor in prostate cancer, but it appears to exert varied biological activity in different diseases. Increasing evidence has indicated that an imbalance in lncRNA DRAIC expression is involved in many diseases especially cancers, including prostate cancer (14–18), lung cancer (19–21), glioma (22–24), breast cancer (25–27), colorectal cancer (28), esophageal cancer (29), gastric cancer (30), nasopharyngeal carcinoma (31), retinoblastoma (32), in addition to Hirschsprung’s disease (33, 34) and omphalocele (35). Abnormal expression levels of DRAIC have also been associated with clinicopathological features of patients, such as lymph node metastasis, neoplasm stage, overall survival and progression-free survival. More notably, lncRNA DRAIC exhibited a vital influence on the modulation of abnormal cellular processes and tumorigenesis progression through cell proliferation, invasion, migration, and autophagy. Mechanistic investigations have further prompted major advances in the clinical applications of lncRNA DRAIC, including its potential for diagnosis, prognosis, and treatment. In this review, we first focus on the biological functions, relevant mechanisms, and future clinical applications of lncRNA DRAIC, and summarize available knowledge on the expression profiles and clinical characteristics of lncRNA DRAIC in disease processes.

## The role of the lncrna draic in cancers

**Figure 1:**
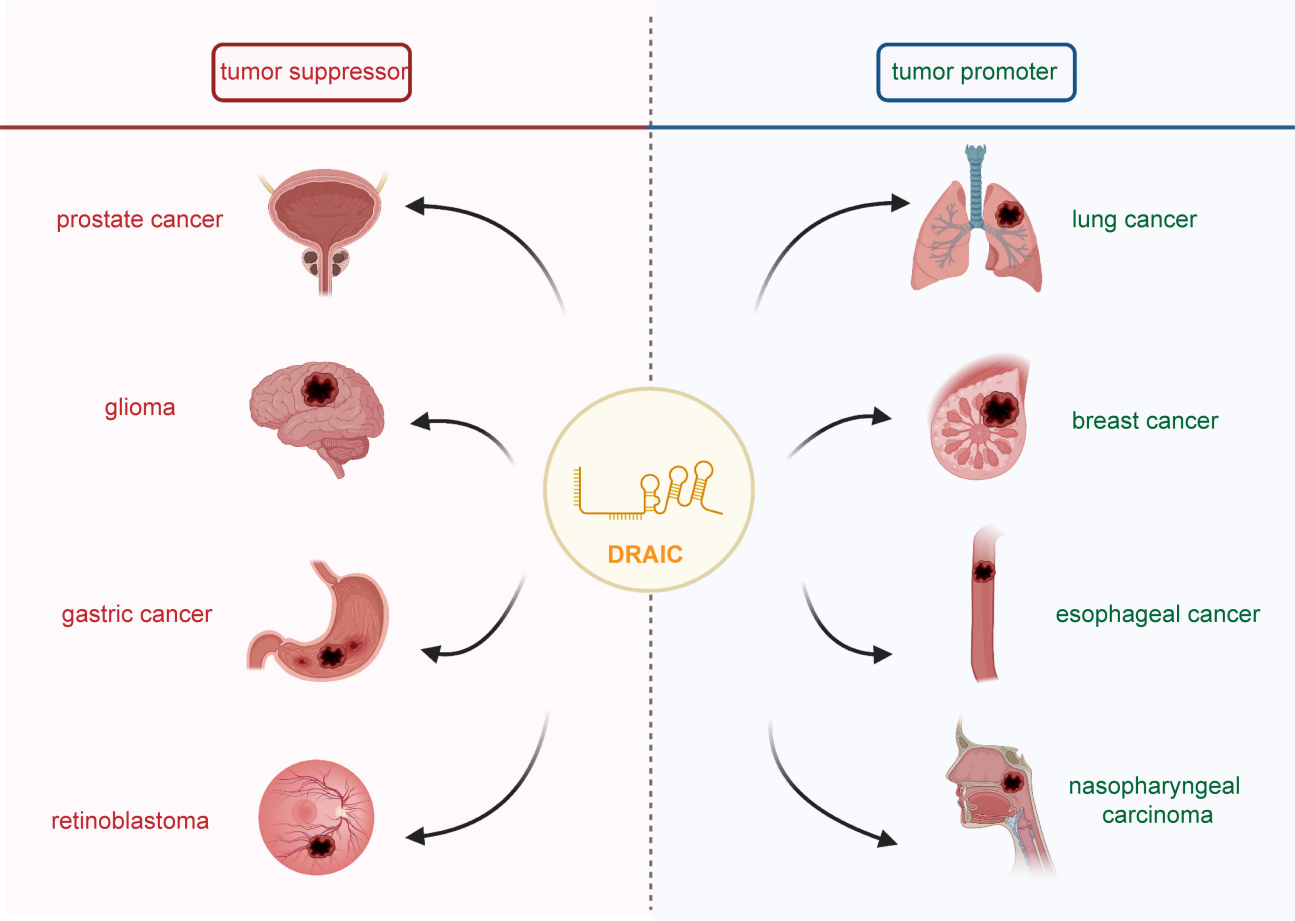
The role of lncRNA DRAIC in human cancers. It has been shown that lncRNA DRAIC acts as a tumor suppressor in prostate cancer, glioma, gastric cancer, and retinoblastoma. DRAIC also functioned as an oncogene in lung cancer, breast cancer, esophageal cancer and nasopharyngeal carcinoma.

LncRNA DRAIC was shown to be aberrantly expressed in several types of human disease, including prostate cancer, lung cancer, glioma, breast cancer, colorectal cancer, esophageal cancer, gastric cancer, nasopharyngeal carcinoma, retinoblastoma, Hirschsprung’s disease, and omphalocele (**Figure 1**). Indeed, lncRNA DRAIC expression was shown to have a significant association with patient clinicopathological features (**Table 1**). LncRNA DRAIC also exerts key roles in multiple cellular processes *via* diverse mechanisms (**Table 2**).

**Table 1:** lncRNA DRAIC expression and clinical characteristics in human diseases.

Disease type	Expression	Clinical characteristics	Refs
prostate cancer	downregulated	overall survival, and disease-free survival	33430890,31900260, 28241429,27562825,25700553
lung cancer	upregulated	TNM stage, lymph node metastasis, and poor prognosis	34764698,34306024,33771173
glioma	downregulated	overall survival, and progression-free survival	34746949,33767991,33336743
breast cancer	upregulated	overall survival, and disease specific survival	34645975,30872794,30544991
esophageal cancer	upregulated	/	32659236
gastric cancer	downregulated	lymph node metastasis	32351584
nasopharyngeal carcinoma	upregulated	advanced clinical stage	31497998
retinoblastoma	downregulated	/	31058073
Hirschsprung’s disease	upregulated	/	34471485,31647312
Omphalocele	downregulated	/	30538881

**Table 2:** Functions and mechanisms of lncRNA DRAIC in cancers.

Disease type	Cell lines	Functions	Related mechanisms	Refs
prostate cancer	LNCaP, and C4-2B cells	cell migration, and invasion	FOXA1, NKX3-1, IKK, and NF-κB	33430890,31900260, 28241429,27562825, 25700553
lung cancer	Calu-3, HCC827, NCI-H441, and NCI-H1975 cells	cell proliferation, migration, and invasion	miR-3940-3p	34764698,34306024, 33771173
glioma	U251, A172, U87, and U373 cells	cell proliferation, migration, invasion, and autophagy	AMPK, NF-κB, mTOR, S6K1, H3K4me3, SET7/9, and miR-18a-3p	34746949,33767991, 33336743
breast cancer	HeLa, T47D, MCF-7, SKBR3, MDA-MB-361, and MDA-MB-231 cells	cell proliferation, migration, invasion, autophagy, and apoptosis	FOXP3, miR-432-5p, and SLBP	34645975,30872794, 30544991
esophageal cancer	Eca-109, TE-1, EC9706, and OE19 cells	cell proliferation, invasion, apoptosis, and autophagy	miR-149-5p, and NFIB	32659236
gastric cancer	HGC-27, SGC-7901, BGC-823, AGS, and MKN45 cells	cell proliferation, migration, and invasion	UCHL5, and NFRKB	32351584
nasopharyngeal carcinoma	CNE-1, and C666-1 cells	cell proliferation, migration, and invasion	miR-122, and SATB1	31497998
retinoblastoma	Y79 cells	cell proliferation	/	31058073

## The tumor-suppressor role of DRAIC in cancers

### Prostate cancer

Prostate cancer (PCa) is the most frequent malignant tumor and accounts for the second leading cause of cancer-related deaths in men (36–40). The androgen receptor (AR) plays a crucial role in the pathogenesis of PCa and is considered a clinically validated target for the treatment of PCa (41–44). Unfortunately, long-term androgen deprivation can ultimately lead to castration-resistant PCa (CRPC), which favors metastasis and poor prognosis (45–47). Although much effort has been made to improve PCa treatment, it is still needed to identify more sensitive biomarkers to guide early diagnosis and treatment (37, 48, 49). Several studies have shown that lncRNA DRAIC is dysregulated in PCa LNCaP and C4-2B cells as well as in 7 PCa tumor biopsies by androgens in a dose and time-dependent manner (14–18). Moreover, lncRNA DRAIC was considered to be a tumor suppressor by preventing the transformation of cuboidal epithelial cells to fibroblast-like morphology as well as cell migration and invasion. *In vivo*, lncRNA prevents the growth of xenograft tumors.

### Glioma

Glioma is one of the most prevalent primary malignant tumors in the central nervous system, accounting for about 81% of malignant brain tumors (50–52). lncRNA DRAIC has been shown to be downregulated in glioma tissues and cell lines (U251, A172, U87, and U373 cells) (22–24). Survival analysis has indicated that a high lncRNA DRAIC expression was associated with a remarkably favorable overall survival and progression-free survival of lower-grade glioma patients who had been submitted to radiotherapy (23). lncRNA DRAIC repressed cell proliferation, migration, invasion, and *in vivo* xenograft tumor growth, as well as induced cell autophagy in U251, A172, and U87 cells (22, 24).

### Gastric cancer

Gastric cancer is one of the most frequent digestive tract cancers, which accounts for a large proportion of cancer-related morbidity and mortality worldwide (53–57). Although advances have been made in the treatment of patients with gastric cancer over the past few years, their 5-year survival rate is still lower than 25% (58–61). Of note, novel biomarkers should be identified to improve the early diagnosis and survival rates of gastric cancer patients (61–63). The expression of lncRNA DRAIC was downregulated according to tumor progression in 67 primary gastric cancer patients who were submitted to surgical resection as well as in HGC-27, SGC-7901, BGC-823, AGS and MKN45 cell lines (30). A high lncRNA DRAIC level was significantly associated with lymph node metastasis, while the downregulation of DRAIC inhibited cell proliferation and metastasis in HGC-27, MKN45, and SGC-7901 cells.

### Pediatric retinoblastoma

Retinoblastoma is the most common intraocular tumor in children and is initiated by the biallelic inactivation of the retinoblastoma 1 (RB1) gene (64–68). A recent a study revealed that lncRNA DRAIC was dysregulated in retinoblastoma Y79 cells and 7 retinoblastoma tissues. This lncRNA was involved in the modulation of Y79 cell growth and proliferation (32).

## The tumor-promoting role of DRAIC in cancers

### Lung cancer

Lung cancer is the most commonly diagnosed malignancy worldwide and lung adenocarcinoma (LUAD) represents the most common histological type of lung cancer (69–73). A late diagnosis of LUAD contributes to high metastasis and mortality rates, emphasizing the urgency for better identification of sensitive biomarkers during lung cancer progression (69, 74–76). High expression of lncRNA DRAIC was recently observed in LUAD tissues and cell lines (Calu-3, HCC827, NCI-H441, and NCI-H1975 cells) and was positively correlated with TNM stage, lymph node metastasis, and a poor prognosis (19–21). lncRNA DRAIC has been proved to exhibit tumorigenic effects through the regulation of cell proliferation, migration, and invasion of Calu-3 and HCC827 cells.

### Breast cancer

Breast cancer is a common malignancy with high incidence and morbidity rates in females (77–81). Therefore, establishing an effective biomarker is essential to decrease mortality and improve the survival rate for breast cancer patients (81–84). lncRNA DRAIC expression was distinctly upregulated in 828 breast cancer specimens and cell lines (HeLa, T47D, MCF-7, SKBR3, MDA-MB-361, and MDA-MB-231 cells). Kaplan–Meier plots and log-rank tests have shown that a high expression of lncRNA DRAIC was correlated with a poorer overall survival and disease specific survival, especially in ER-positive breast cancer patients (27). In addition, lncRNA DRAIC stimulated tumor progression through the promotion of cell proliferation, migration, and invasion, as well as the inhibition of cell autophagy and apoptosis in SKBR3, MCF-7 and MDA-MB-231 cells (25, 26).

### Esophageal cancer

Esophageal cancer is a common upper gastrointestinal malignancy that ranks eighth in the world among cancer incidence, especially in China (85–89). High levels of DRAIC were found in esophageal cancer cells Eca-109, TE-1, EC9706, and OE19 (29). Moreover, DRAIC played an oncogene role since it facilitated cell proliferation and invasion, and repressed cell apoptosis and autophagy in Eca-109 and EC9706 cells.

### Nasopharyngeal carcinoma

Nasopharyngeal carcinoma is an epithelial carcinoma generated within the nasopharyngeal mucosal lining (90–94). lncRNA DRAIC was highly expressed in nasopharyngeal carcinoma cell lines CNE-1 and C666-1 as well as in 32 biopsy tissues (31). Moreover, a high expression level of lncRNA DRAIC showed a close relationship with higher clinical stages. In addition, lncRNA DRAIC acted as an oncogene and enhanced cell proliferation, migration and invasion in CNE-1 and C666-1 cells.

Accumulating evidence has reported that the differential expression of DRAIC in prostate cancer, lung cancer, glioma, breast cancer, colorectal cancer, esophageal cancer, gastric cancer, nasopharyngeal carcinoma, and retinoblastoma. And its abnormal expression was significantly related to many clinicopathological features, notably the patient’s prognosis. Furthermore, DRAIC was implicated as a regulator of a wide variety of cellular processes and then participated in the pathogenesis and progression of numerous human disorders. Therefore, elucidating the underlying molecular mechanisms of DRAIC in cancer progression has been proven to hold promise to support its clinical application significance.

## Regulatory mechanisms of lncrana draic

Several studies have reported that lncRNA DRAIC actively participates in crucial biological processes of many diseases, such as cell proliferation, apoptosis, autophagy, invasion and migration. Here, we discuss the main biological functions and molecular mechanisms of lncRNA DRAIC during disease progression.

### Cell proliferation

It is well known that cells proliferate excessively which ultimately results in tumor progression (95–98). In glioma, lncRNA DRAIC has been demonstrated to suppress the proliferation of U251 cells by targeting miR-18a-3p (24). And lncRNA DRAIC was activated by FOXP3 in breast cancer and promoted cell proliferation in SKBR3 and MDA-MB-231 cells *via* sponging miR-432-5p to increase SLBP levels (25). lncRNA DRAIC was also found to improve MCF-7 cell proliferation in an autophagy-independent manner by regulating the activity of ULK1 and enhancing LC3B expression (26). Similarly, lncRNA DRAIC led to cell proliferation in esophageal cancer Eca-109 and EC9706 cells through the miR-149-5p/NFIB axis (29). In gastric cancer, lncRNA DRAIC has also been indicated to inhibit the proliferation of SGC-7901, HGC-27, and MKN45 cells by binding to UCHL5 and accelerating the ubiquitination of NFRKB (30). Additionally, lncRNA DRAIC increased the proliferation of nasopharyngeal carcinoma CNE-1 and C666-1 cells *via* an interaction with miR-122 and up-regulation of SATB1 (31).

### Cell migration and invasion

Metastasis, also termed invasion-migration cascade, is a multistep process that involves the dissemination of tumor cells from the primary tumor site to distant organs and subsequent formation of secondary tumors (99–103). As the major reason behind most cancer-related deaths, metastasis is a current challenge to improve the survival of cancer patients (99, 104–107).

**Figure 2:**
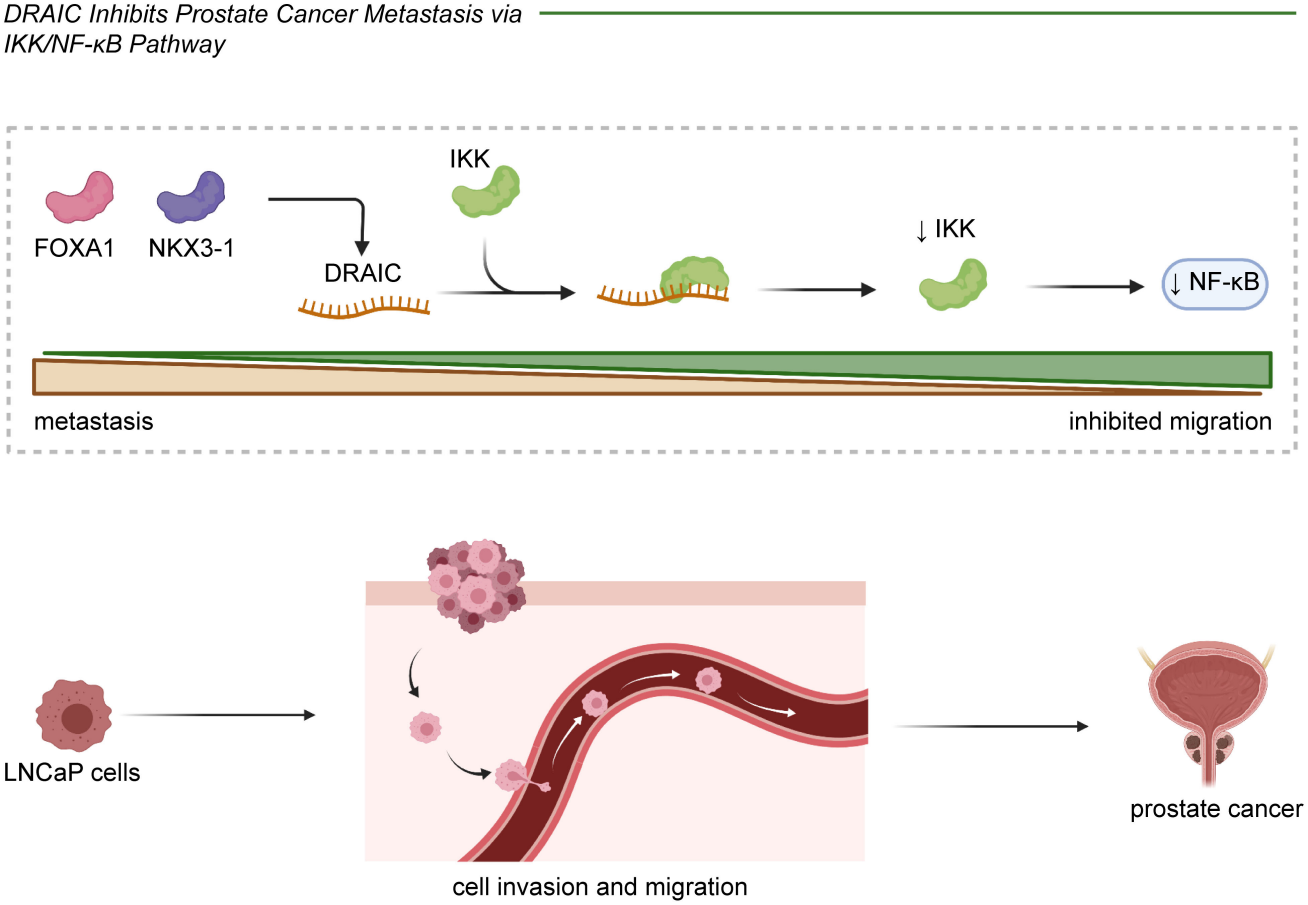
In prostate cancer, lncRNA DRAIC played a tumor suppressive role through inhibiting cell migration and invasion. DRAIC was activated by FOXA1 and NKX3-1 and interacts with IKK to further decrease NF-κB expression in LNCaP cells.

DRAIC was shown to positively regulate FOXA1 and NKX3-1 and to block the transformation of LNCaP prostate cancer cuboidal epithelial cells to a fibroblast-like morphology. This subsequently hindered cell migration and invasion through an interaction with IKK that inactivated NF-κB (**Figure 2**) (14, 16). In glioma cells, lncRNA DRAIC also exerted pro-migratory and invasive roles *via* the repression of NF-κB, coupled with increases in AMPK phosphorylation and thus inhibition of mTOR activity and phosphorylation of key substrates like S6K1 (22). Moreover, the interaction between the H3K4me3 protein and the lncRNA DRAIC promoter was mediated by SET7/9 and increased the association of DRAIC with miR-18a-3p. These mechanisms were shown to improve the metastasis of U251 cells (24). And in breast cancer cell lines, lncRNA DRAIC was up-regulated by FOXP3 and promoted cell migration and invasion *via* the miR-432-5p/SLBP axis (25).Moreover, lncRNA DRAIC improved esophageal cancer Eca-109 and EC9706 cell invasion through binding to miR-149-5p, which regulated NFIB levels (29). lncRNA DRAIC also hindered cell metastasis through its interaction with UCHL5 and repression of NFRKB deubiquitination in gastric cancer (30). In nasopharyngeal carcinoma cells, lncRNA DRAIC boosted cell migration and invasion through its interaction with miR-122 and the consequent increase of SATB1 levels (31). Furthermore, lncRNA DRAIC facilitated the migration of HSCR 293T and SH-SY5Y cells by sponging miR-34a-5p, which positively modulated ITGA6 expression (33).

### Cell autophagy

**Figure 3:**
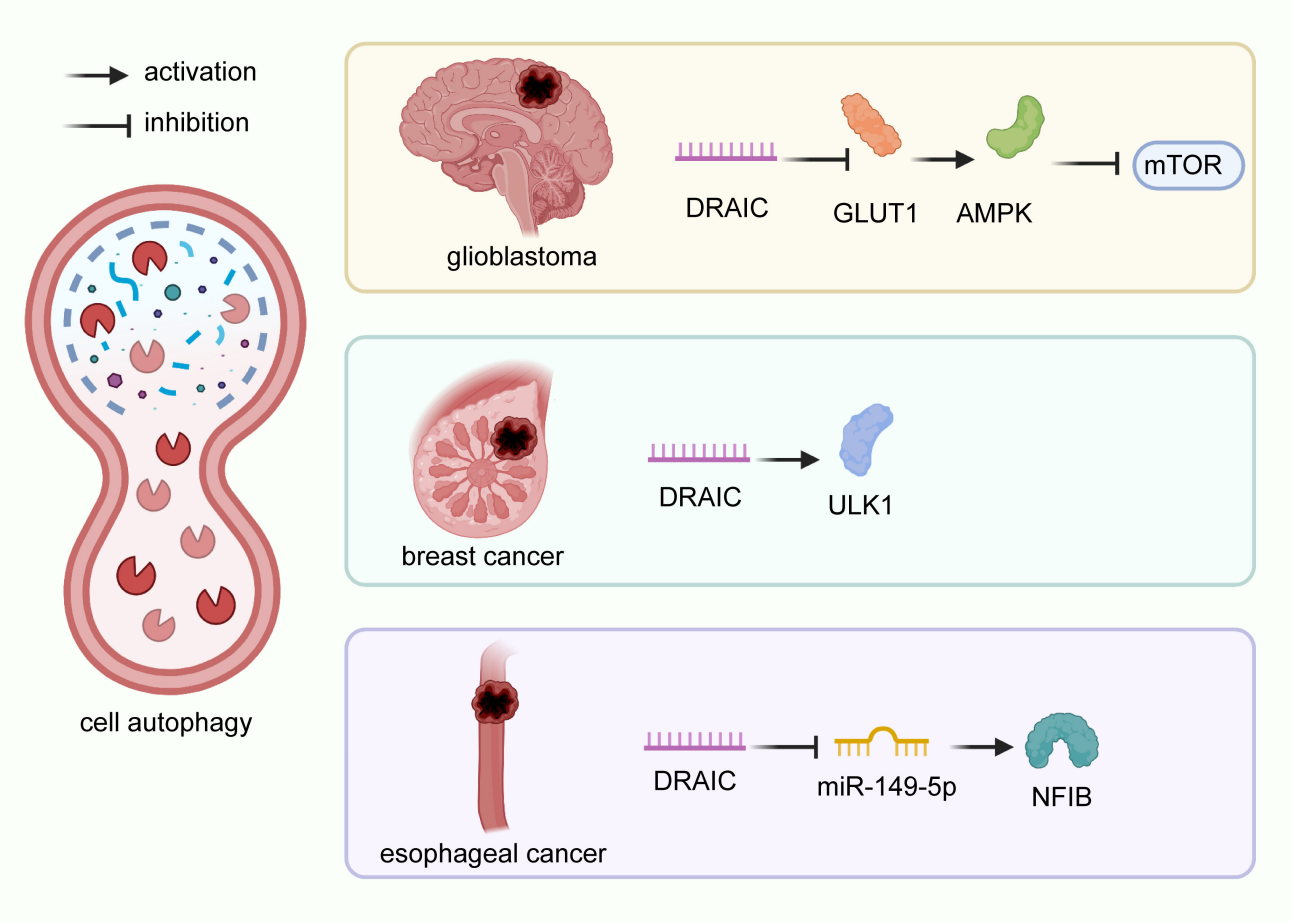
Regulatory mechanisms of DRAIC on cell autophagy in cancers. In glioblastoma A172 and U87 cells, lncRNA DRAIC induced autophagy by downregulating the NF-κB target gene GLUT1, which activated AMPK, and blocked the expression of mTOR. In breast cancer MCF-7 cells, DRAIC enhanced ULK1 expression and inhibited cell autophagy. In esophageal cancer, lncRNA DRAIC suppressed cell autophagy through its interaction with miR-149-5p and up-regulation of NFIB.

**Figure 4:**
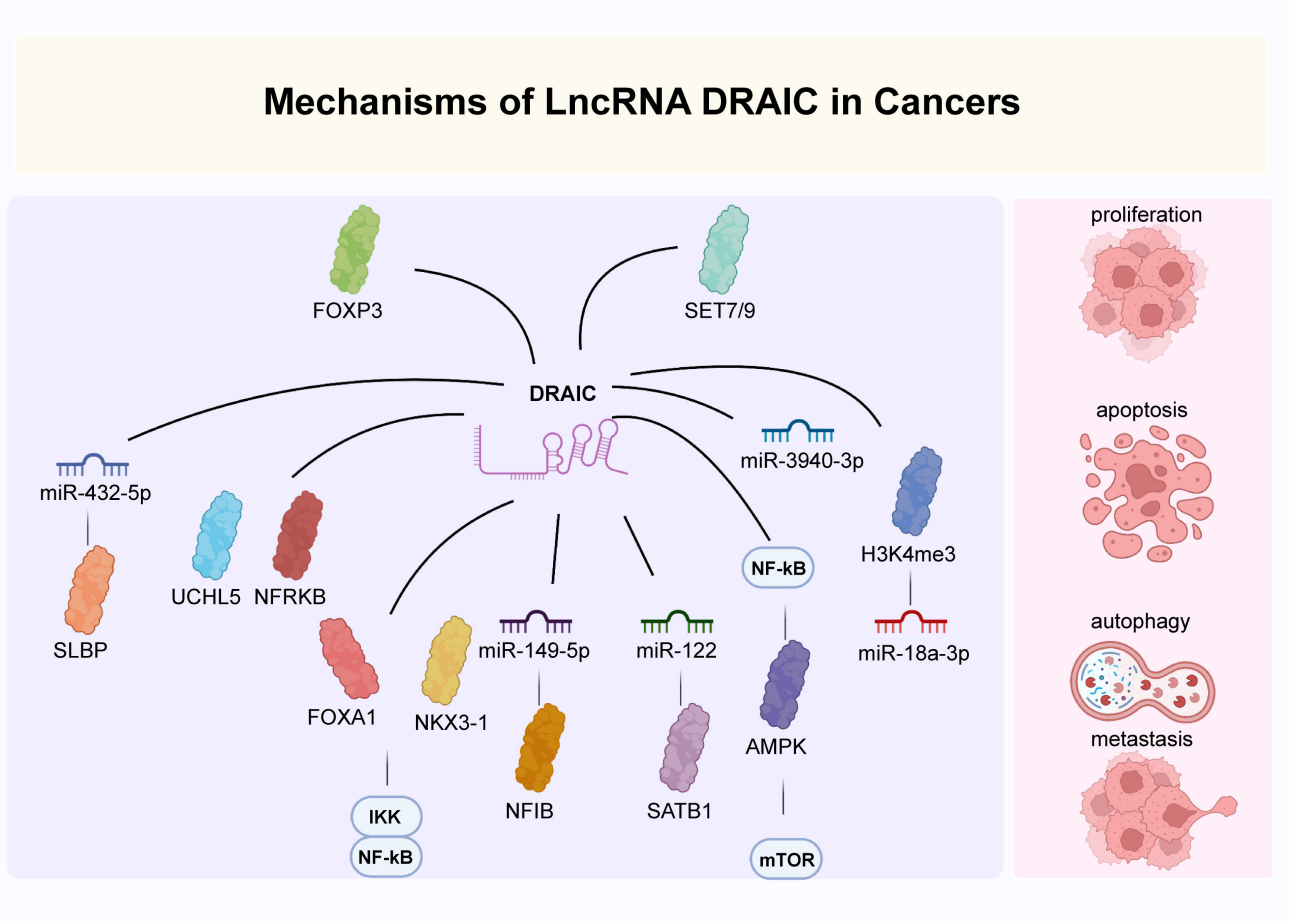
The main mechanisms of DRAIC in cancers. DRAIC participated in the regulation of biological processes of cancers through interaction with diverse molecules.

Autophagy is a process of intracellular component degradation that maintains cellular homeostasis (26, 108–111). Its dysfunction contributes to a series of pathophysiological processes of various diseases, including cancer. Studies have identified that numerous lncRNAs regulate autophagy through various mechanisms (112–115). lncRNA DRAIC has been reported modeulate autophagy in glioblastoma A172 and U87 cells by downregulating the NF-κB target gene GLUT1, increasing AMPK levels, and thus inhibiting mTOR (22, 116). lncRNA DRAIC also suppressed cell autophagy in breast cancer MCF-7 cells through the activation of ULK1 (26). Similarly, lncRNA DRAIC was found to inhibit cell autophagy in esophageal cancer Eca-109 and EC9706 cells acting as ceRNAs to modulate the expression of NFIB by quenching miR-149-5p (**Figure 3**) (29). Take together, DRAIC was involved in the multiple biological process of cancers through interaction with diverse molecules (**Figure 4**).

## Prospects for the clinical applications of draic in disease management

In recent years, numerous studies have shown the significance of lncRNAs in clinical applications for human disease, especially in cancer (117–121). lncRNA DRAIC is a newly identified lncRNA involved in multiple human diseases. Previous evidence suggests that DRAIC is extensively involved in the modulation of numerous biological functions and intimately associated with pathological characteristics, which may be valuable for clinical diagnosis, prognosis, and treatment. In this section, we address the promising significance of lncRNA DRAIC in human disease.

### DRAIC as a diagnostic and prognostic biomarker

The expression levels of lncRNA DRAIC in a diverse array of tissues and cell lines were observed to be differentially regulated depending on the disease state, which reveals that lncRNA DRAIC expression can be used to distinguish between normal and diseased tissues. Therefore, assessing lncRNA DRAIC concentration may effectively act as a method for the early diagnosis of diseases. Besides, increasing data supports that lncRNA DRAIC expression is significantly associated with a variety of clinicopathological features, demonstrating the promising potential for prognosis prediction. For example, lower levels of DRAIC were observed as PCa progressed from AD to CR. This was associated with a lower disease-free-survival rate of patients verified by the Kaplan-Meier plot (14). lncRNA DRAIC was overexpressed at a higher level in high malignancy breast cancers when compared to low malignancy cases, suggesting its diagnostic and prognostic value (27). In low-grade glioma, DRAIC was shown to reflect the prognosis of radiotherapy treatment (27). Additionally, lncRNA DRAIC was perceived as a novel prognosis biomarker for risk evaluation of HSCR (34). In LUAD, lncRNA DRAIC was regarded as an immune-related RNA and incorporated into the 5-lncRNA-based model and 5-lncRNA risk signature, which has been shown to accurately predict the prognosis of patients (20, 21). However, lncRNA DRAIC was mainly measured in cell lines and tissues, which must be optimized to a more accessible and convenient approach. Tissue biopsy has several drawbacks, such as invasiveness, complicated manipulation, high cost, bleeding complications, poor reproducibility, and sampling variability. Minimally invasive (e.g., saliva, urine, and blood) detection of lncRNA DRAIC expression and sensitivity is an increasing research interest for its diagnostic and prognostic applications.

### LncRNA DRAIC as a treatment target

The abnormal expression of lncRNA DRAIC in disease also provided novel insights for disease treatment. Alterations of lncRNA DRAIC expression may be developed as a therapeutic target for the inhibition of disease progression. Furthermore, the molecular mechanisms through which DRAIC regulates the pathogenesis of diseases also resulted in an effective therapy target. Knockdown or activation of lncRNA DRAIC and relevant molecules, as well as the regulation of intramolecular interactions, may also serve as potential targeting candidates for novel pharmaceutical development and molecular-targeted therapies (122). Indeed, lncRNA DRAIC knockout was confirmed to suppress the tumorigenesis of PCa PC3M cells by inhibiting the NF-κB pathway in nude mice. Moreover, lncRNA DRAIC expression was shown to reflect the sensitivity of tumor cells to chemotherapy or radiotherapy. For example, lncRNA DRAIC expression was demonstrated to predict patient response to radiosensitivity in lower-grade glioma (23). In breast cancer, the expression level of lncRNA DRAIC can reflect the efficacy of chemotherapy drugs, such as paclitaxel, FEC, and lapatinib, which may contribute to guiding more sensitized and individualized treatment options for patients (27). In addition, existing research on DRAIC has mainly been concentrated on the cellular level with a deficiency of *in vivo* studies. lncRNA DRAIC was currently explored in only a small portion of human diseases, and there is little known about the multifaceted role and functional mechanisms of DRAIC in other types of disease. Further *in vivo* experiments are required to determine whether the molecular mechanisms of lncRNA DRAIC on disease progression discovered by *in vitro* studies are consistent. Besides, more mechanistic insights of lncRNA DRAIC in other diseases probably also contribute to the development of better-targeted therapeutics.

In general, lncRNA DRAIC was proved to be a potential diagnostic and prognosis biomarker, together with a treatment target for human cancers. Further investigation is needed to determine the expression profile, sensitivity and stability of lncRNA DRAIC in non-invasive samples. This could improve disease diagnosis and prognosis as well as the efficiency and safety of lncRNA DRAIC-targeted treatment.

## Conclusion

Numerous reports have shown that lncRNA DRAIC is abnormally expressed in PCa, lung cancer, glioma, breast cancer, colorectal cancer, esophageal cancer, gastric cancer, nasopharyngeal carcinoma, retinoblastoma, HSRC, and omphalocele. Moreover, lncRNA DRAIC exhibited a significant association with patient clinicopathological characteristics, especially immune cell infiltration, tumor stage, lymph node metastasis, overall survival and progression-free survival. lncRNA DRAIC was demonstrated to exert momentous roles in multiple cellular process, such as cell proliferation, invasion, migration, and autophagy. Functional assays have revealed a series of molecular mechanisms of lncRNA DRAIC in the development of diseases. These features can be exploited for various medicinal applications, including diagnosis, prognosis and treatment of human diseases.

Extensive research has been undertaken to explore the clinical application of lncRNAs in the past few years (123). The majority of non-invasive biopsy biomarkers are currently being investigated for diagnostic and prognostic purposes (124). The detection of lncRNA DRAIC expression can be used as a promising clinical biomarker for early diagnosis and prognosis. Additional studies are necessary to validate whether lncRNA DRAIC can be detected in non-invasive samples and further verify the stability and specificity of its expression in non-invasive samples. Moreover, lncRNA DRAIC and the relevant molecular pathways may also be applied as new candidates for targeted treatment of several diseases. However, the available studies mainly focus on the expression of lncRNA DRAIC on clinical tissue samples and *in-vitro* cell lines, lacking enough *in vivo* animal studies. The follow-up animal experiments and prospective studies are needed to confirm the efficacy and safety of lncRNA DRAIC-targeted therapy. And the roles and mechanisms of lncRNA DRAIC have merely been explored in a comparably small number of diseases. It is necessary to further probe the role of lncRNA DRAIC in other disease types.

## Author contributions

DC provided a source of ideas for this review. XZ collected the related paper. QY drafted and reviewed the manuscript. All authors have contributed substantially to the original research and approved the submitted version.

## Funding

This work was funded by the National Nature Science Foundation (81802085).

## Conflict of interest

The authors declare that the research was conducted in the absence of any commercial or financial relationships that could be construed as a potential conflict of interest.

## Publisher’s note

All claims expressed in this article are solely those of the authors and do not necessarily represent those of their affiliated organizations, or those of the publisher, the editors and the reviewers. Any product that may be evaluated in this article, or claim that may be made by its manufacturer, is not guaranteed or endorsed by the publisher.

## References

[1] Huang X Sun L Wen S Deng D Wan F He X. RNA Sequencing of plasma exosomes revealed novel functional long noncoding RNAs in hepatocellular carcinoma. Cancer Sci (2020) 9:3338–49. doi: 10.1111/cas.14516 PMC7469810 32506598

[2] Zhou Q Hou Z Zuo S Zhou X Feng Y Sun Y. LUCAT1 promotes colorectal cancer tumorigenesis by targeting the ribosomal protein L40-MDM2-p53 pathway through binding with UBA52. Cancer Sci (2019) 4:1194–207. doi: 10.1111/cas.13951 PMC6447850 30690837

[3] Braicu C Zimta AA Harangus A Iurca I Irimie A Coza O. The function of non-coding RNAs in lung cancer tumorigenesis. Cancers (Basel) (2019) 5:605. doi: 10.3390/cancers11050605 PMC6563001 31052265

[4] Lu W Cao F Wang S Sheng X Ma J. LncRNAs: The regulator of glucose and lipid metabolism in tumor cells. Front Oncol (1099) 2019). doi: 10.3389/fonc.2019.01099 PMC6901916 31850189

[5] Pan H Ding Y Jiang Y Xia X Ji Y. LncRNA LIFR-AS1 promotes proliferation and invasion of gastric cancer cell *via* miR-29a-3p/COL1A2 axis. Cancer Cell Int (2021) 1:7. doi: 10.1186/s12935-020-01644-7 PMC7789183 33407453

[6] Yang YT Wang YF Lai JY Ming X Tu YF Tian J. Long non-coding RNA UCA1 contributes to the progression of oral squamous cell carcinoma by regulating the WNT/β-catenin signaling pathway. Cancer Sci (2016) 11:1581–9. doi: 10.1111/cas.13058 PMC5132283 27560546

[7] Sun W Nie W Wang Z Zhang H Li Y Fang X. Lnc HAGLR promotes colon cancer progression through sponging miR-185-5p and activating CDK4 and CDK6 *in vitro* and in vivo. Onco Targets Ther (2020) 13:5913–25. doi: 10.2147/OTT.S246092 PMC7319508 32606801

[8] Parfenyev S Singh A Fedorova O Daks A Kulshreshtha R Barlev NA. Interplay between p53 and non-coding RNAs in the regulation of EMT in breast cancer. Cell Death Dis (2021) 1:17. doi: 10.1038/s41419-020-03327-7 PMC7791039 33414456

[9] Hu X Goswami S Qiu J Chen Q Laverdure S Sherman BT. Profiles of long non-coding RNAs and mRNA expression in human macrophages regulated by interleukin-27. Int J Mol Sci (2019) 24:6207. doi: 10.3390/ijms20246207 PMC6941108 31835347

[10] Prensner JR Chinnaiyan AM. The emergence of lncRNAs in cancer biology. Cancer Discov (2011) 5:391–407. doi: 10.1158/2159-8290.CD-11-0209 PMC3215093 22096659

[11] Ulitsky I Bartel DP. lincRNAs: genomics, evolution, and mechanisms. Cell (2013) 1:26–46. doi: 10.1016/j.cell.2013.06.020 PMC3924787 23827673

[12] Wang LQ Zheng YY Zhou HJ Zhang XX Wu P Zhu SM. LncRNA-fendrr protects against the ubiquitination and degradation of NLRC4 protein through HERC2 to regulate the pyroptosis of microglia. Mol Med (Cambridge Mass) (2021) 1:39. doi: 10.1186/s10020-021-00299-y PMC8048261 33858325

[13] Li X Wu Z Fu X Han W. lncRNAs: insights into their function and mechanics in underlying disorders. Mutat Res Rev Mutat Res (2014) 762:1–21. doi: 10.1016/j.mrrev.2014.04.002 25485593

[14] Sakurai K Reon BJ Anaya J Dutta A. The lncRNA DRAIC/PCAT29 locus constitutes a tumor-suppressive nexus. Mol Cancer Res (2015) 5:828–38. doi: 10.1158/1541-7786.MCR-15-0016-T PMC4456356 25700553

[15] Videira A Beckedorff FC daSilva LF Verjovski-Almeida S. PVT1 signals an androgen-dependent transcriptional repression program in prostate cancer cells and a set of the repressed genes predicts high-risk tumors. Cell Commun Signal (2021) 1:5. doi: 10.1186/s12964-020-00691-x PMC7798249 33430890

[16] Saha S Kiran M Kuscu C Chatrath A Wotton D Mayo MW. Long noncoding RNA DRAIC inhibits prostate cancer progression by interacting with IKK to inhibit NF-κB activation. Cancer Res (2020) 5:950–63. doi: 10.1158/0008-5472.CAN-19-3460 PMC7056510 31900260

[17] Smolle MA Bauernhofer T Pummer K Calin GA Pichler M. Current insights into long non-coding RNAs (LncRNAs) in prostate cancer. Int J Mol Sci (2017) 2:473. doi: 10.3390/ijms18020473 PMC5344005 28241429

[18] Colditz J Rupf B Maiwald C Baniahmad A. Androgens induce a distinct response of epithelial-mesenchymal transition factors in human prostate cancer cells. Mol Cell Biochem (2016) 1-2:139–47. doi: 10.1007/s11010-016-2794-y 27562825

[19] Liu Z Yang S Zhou S Dong S Du J. Prognostic value of lncRNA DRAIC and miR-3940-3p in lung adenocarcinoma and their effect on lung adenocarcinoma cell progression. Cancer Manage Res (2021) 13:8367–76. doi: 10.2147/CMAR.S320616 PMC8577463 34764698

[20] Wu G Wang Q Zhu T Fu L Li Z Wu Y. Identification and validation of immune-related LncRNA prognostic signature for lung adenocarcinoma. Front Genet (2021) 12:681277. doi: 10.3389/fgene.2021.681277 34306024 PMC8301374

[21] Mu L Ding K Tu R Abounader R Dutta A. Identification of 4 immune cells and a 5-lncRNA risk signature with prognosis for early-stage lung adenocarcinoma. J Trans Med (2021) 1:127. doi: 10.1186/s12967-021-02800-x PMC8004399 33771173

[22] Saha S Zhang Y Wilson B Abounader R Dutta A. The tumor-suppressive long noncoding RNA DRAIC inhibits protein translation and induces autophagy by activating AMPK. J Cell Sci (2021) 24:jcs259306. doi: 10.1242/jcs.259306 PMC8729785 34746949

[23] Li Z Cai S Li H Gu J Tian Y Cao J. Developing a lncRNA signature to predict the radiotherapy response of lower-grade gliomas using Co-expression and ceRNA network analysis. Front Oncol (2021) 11:622880. doi: 10.3389/fonc.2021.622880 33767991 PMC7985253

[24] Li C Feng SY Chen L. SET7/9 promotes H3K4me3 at lncRNA DRAIC promoter to modulate growth and metastasis of glioma. Eur Rev Med Pharmacol Sci (2020) 23:12241–50. doi: 10.26355/eurrev_202012_24016 33336743

[25] Li S Jia H Zhang Z Wu D. DRAIC promotes growth of breast cancer by sponging miR-432-5p to upregulate SLBP. Cancer Gene Ther (2021) 29:951–60. doi: 10.1038/s41417-021-00388-4 34645975

[26] Tiessen I Abildgaard MH Lubas M Gylling HM Steinhauer C Pietras EJ. A high-throughput screen identifies the long non-coding RNA DRAIC as a regulator of autophagy. Oncogene (2019) 26:5127–41. doi: 10.1038/s41388-019-0783-9 30872794

[27] Zhao D Dong JT. Upregulation of long non-coding RNA DRAIC correlates with adverse features of breast cancer. Noncoding RNA (2018) 4:39. doi: 10.3390/ncrna4040039 PMC6315495 30544991

[28] Zhang C Wang Y Wang P Jiang L Xiao X. LncRNA DRAIC promotes apoptosis and inhibits proliferation of colorectal cancer *via* regulating MiR-223. Minerva Med (2021) 10.23736:S0026–4806. doi: 10.23736/S0026-4806.21.07605-9 34142774

[29] Li F Zhou X Chen M Fan W. Regulatory effect of LncRNA DRAIC/miR-149-5p/NFIB molecular network on autophagy of esophageal cancer cells and its biological behavior. Exp Mol Pathol (2020) 116:104491. doi: 10.1016/j.yexmp.2020.104491 32659236

[30] Zhang Z Hu X Kuang J Liao J Yuan Q. LncRNA DRAIC inhibits proliferation and metastasis of gastric cancer cells through interfering with NFRKB deubiquitination mediated by UCHL5. Cell Mol Biol Lett (2020) 25:29. doi: 10.1186/s11658-020-00221-0 32351584 PMC7183705

[31] Liao B Wang Z Zhu Y Wang M Liu Y. Long noncoding RNA DRAIC acts as a microRNA-122 sponge to facilitate nasopharyngeal carcinoma cell proliferation, migration and invasion *via* regulating SATB1. Artif Cells Nanomed Biotechnol (2019) 1:3585–97. doi: 10.1080/21691401.2019.1656638 31497998

[32] Rajasekaran S Nagarajha Selvan LD Dotts K Kumar R Rishi P Khetan V. Non-coding and coding transcriptional profiles are significantly altered in pediatric retinoblastoma tumors. Front Oncol (2019) 221. doi: 10.3389/fonc.2019.00221 PMC6477087 31058073

[33] Sun C Xu B Wang L Su Y. LncRNA DRAIC regulates cell proliferation and migration by affecting the miR-34a-5p/ITGA6 signal axis in hirschsprung’s disease. Ups J Med Sci (2021) 126:10.48101. doi: 10.48101/ujms.v126.7895 PMC8383934 34471485

[34] Niu X Xu Y Gao N Li A. Weighted gene coexpression network analysis reveals the critical lncRNAs and mRNAs in development of hirschsprung’s disease. J Comput Biol (2020) 7:1115–29. doi: 10.1089/cmb.2019.0261 31647312

[35] Zhou HF O’Conor CJ Gangahar C Dehner LP. 15q23 gain in a neonate with a giant omphalocele and multiple Co-occurring anomalies. Case Rep Pediatr (2018) 2018:8702568. doi: 10.1155/2018/8702568 30538881 PMC6257893

[36] Masato M Miyata Y Kurata H Ito H Mitsunari K Asai A. Oral administration of e-type prostanoid (EP) 1 receptor antagonist suppresses carcinogenesis and development of prostate cancer *via* upregulation of apoptosis in an animal model. Sci Rep (2021) 1:20279. doi: 10.1038/s41598-021-99694-y PMC8514456 34645904

[37] Zhou J Wang H Cannon V Wolcott KM Song H Yates C. Side population rather than CD133(+) cells distinguishes enriched tumorigenicity in hTERT-immortalized primary prostate cancer cells. Mol Cancer (2011) 10:112. doi: 10.1186/1476-4598-10-112 21917149 PMC3180433

[38] Torre LA Siegel RL Ward EM Jemal A. Global cancer incidence and mortality rates and trends–an update. Cancer Epidemiol Biomarkers Prev (2016) 1:16–27. doi: 10.1158/1055-9965.EPI-15-0578 26667886

[39] Conte A Kisslinger A Procaccini C Paladino S Oliviero O de Amicis F. Convergent effects of resveratrol and PYK2 on prostate cells. Int J Mol Sci (2016) 9:1542. doi: 10.3390/ijms17091542 PMC5037816 27649143

[40] T JMC Arif M Niessen WJ Schoots IG Veenland JF. Automated classification of significant prostate cancer on MRI: A systematic review on the performance of machine learning applications. Cancers (Basel) (2020) 6:1606. doi: 10.3390/cancers12061606 PMC7352160 32560558

[41] Li Q Deng Q Chao HP Liu X Lu Y Lin K. Linking prostate cancer cell AR heterogeneity to distinct castration and enzalutamide responses. Nat Commun (2018) 1:3600. doi: 10.1038/s41467-018-06067-7 PMC6127155 30190514

[42] Baumgart SJ Nevedomskaya E Lesche R Newman R Mumberg D Haendler B. Darolutamide antagonizes androgen signaling by blocking enhancer and super-enhancer activation. Mol Oncol (2020) 9:2022–39. doi: 10.1002/1878-0261.12693 PMC7463324 32333502

[43] Shiota M Fujimoto N Kashiwagi E Eto M. The role of nuclear receptors in prostate cancer. Cells (2019) 6:602. doi: 10.3390/cells8060602 PMC6627805 31212954

[44] Schneider JA Craven TW Kasper AC Yun C Haugbro M Briggs EM. Design of peptoid-peptide macrocycles to inhibit the β-catenin TCF interaction in prostate cancer. Nat Commun (2018) 1:4396. doi: 10.1038/s41467-018-06845-3 PMC6199279 30352998

[45] Song J Chen W Zhu G Wang W Sun F Zhu J. Immunogenomic profiling and classification of prostate cancer based on HIF-1 signaling pathway. Front Oncol (2020) 1374. doi: 10.3389/fonc.2020.01374 PMC7425731 32850440

[46] Ma Y Fan B Ren Z Liu B Wang Y. Long noncoding RNA DANCR contributes to docetaxel resistance in prostate cancer through targeting the miR-34a-5p/JAG1 pathway. Onco Targets Ther (2019) 12:5485–97. doi: 10.2147/OTT.S197009 PMC6636610 31371987

[47] Takayama KI Kosaka T Suzuki T Hongo H Oya M Fujimura T. Subtype-specific collaborative transcription factor networks are promoted by OCT4 in the progression of prostate cancer. Nat Commun (2021) 1:3766. doi: 10.1038/s41467-021-23974-4 PMC8213733 34145268

[48] Abou D Benabdallah N Jiang W Peng L Zhang H Villmer A. Prostate cancer theranostics - an overview. Front Oncol (2020) 884. doi: 10.3389/fonc.2020.00884 PMC7290246 32582550

[49] Qian Y Zhang L Cai M Li H Xu H Yang H. The prostate cancer risk variant rs55958994 regulates multiple gene expression through extreme long-range chromatin interaction to control tumor progression. Sci Adv (2019) 7:eaaw6710. doi: 10.1126/sciadv.aaw6710 PMC6636982 31328168

[50] Louis DN Ohgaki H Wiestler OD Cavenee WK Burger PC Jouvet A. The 2007 WHO classification of tumours of the central nervous system. Acta Neuropathol (2007) 2:97–109. doi: 10.1007/s00401-007-0243-4 PMC1929165 17618441

[51] Weller M Wick W Aldape K Brada M Berger M Pfister SM. Glioma. Nat Rev Dis Primers (2015) 1:15017. doi: 10.1038/nrdp.2015.17 27188790

[52] Ostrom QT Cioffi G Gittleman H Patil N Waite K Kruchko C. CBTRUS statistical report: Primary brain and other central nervous system tumors diagnosed in the united states in 2012-2016. Neuro Oncol (2019) Suppl 5:v1–v100. doi: 10.1093/neuonc/noz150 PMC6823730 31675094

[53] Sun J Wang X Zhang Z Zeng Z Ouyang S Kang W. The sensitivity prediction of neoadjuvant chemotherapy for gastric cancer. Front Oncol (2021) 11:641304. doi: 10.3389/fonc.2021.641304 33937042 PMC8085495

[54] Maruyama R Akino K Toyota M Suzuki H Imai T Ohe-Toyota M. Cytoplasmic RASSF2A is a proapoptotic mediator whose expression is epigenetically silenced in gastric cancer. Carcinogenesis (2008) 7:1312–8. doi: 10.1093/carcin/bgn060 PMC2500213 18310659

[55] Yang Y Wu F Zhang J Sun R Li F Li Y. EGR1 interacts with DNMT3L to inhibit the transcription of miR-195 and plays an anti-apoptotic role in the development of gastric cancer. J Cell Mol Med (2019) 11:7372–81. doi: 10.1111/jcmm.14597 PMC6815817 31515938

[56] Bray F Ferlay J Soerjomataram I Siegel RL Torre LA Jemal A. Global cancer statistics 2018: GLOBOCAN estimates of incidence and mortality worldwide for 36 cancers in 185 countries. CA Cancer J Clin (2018) 6:394–424. doi: 10.3322/caac.21492 30207593

[57] Van Cutsem E Sagaert X Topal B Haustermans K Prenen H. Gastric cancer. Lancet (Lond Engl) (2016) 10060:2654–64. doi: 10.1016/S0140-6736(16)30354-3 27156933

[58] Hayakawa Y Fox JG Gonda T Worthley DL Muthupalani S Wang TC. Mouse models of gastric cancer. Cancers (Basel) (2013) 1:92–130. doi: 10.3390/cancers5010092 PMC3730302 24216700

[59] Zhao CM Hayakawa Y Kodama Y Muthupalani S Westphalen CB Andersen GT. Denervation suppresses gastric tumorigenesis. Sci Trans Med (2014) 250:250ra115. doi: 10.1126/scitranslmed.3009569 PMC4374618 25143365

[60] Fu DJ Wang L Chouairi FK Rose IM Abetov DA Miller AD. Gastric squamous-columnar junction contains a large pool of cancer-prone immature osteopontin responsive Lgr5(-)CD44(+) cells. Nat Commun (2020) 1:84. doi: 10.1038/s41467-019-13847-2 PMC6941991 31901081

[61] Ye D Li Y Zhang H Zhou Z Tang Y Wu P. Silencing PRSS1 suppresses the growth and proliferation of gastric carcinoma cells *via* the ERK pathway. Int J Biol Sci (2021) 4:957–71. doi: 10.7150/ijbs.52591 PMC8040304 33867821

[62] Cao G Li P He X Jin M Li M Chen S. FHL3 contributes to EMT and chemotherapy resistance through up-regulation of slug and activation of TGFβ/Smad-independent pathways in gastric cancer. Front Oncol (2021) 11:649029. doi: 10.3389/fonc.2021.649029 34150617 PMC8213027

[63] Yang X Zhang Z Zhang L Zhou L. MicroRNA hsa-mir-3923 serves as a diagnostic and prognostic biomarker for gastric carcinoma. Sci Rep (2020) 1:4672. doi: 10.1038/s41598-020-61633-8 PMC7070044 32170105

[64] Wei R Ren X Kong H Lv Z Chen Y Tang Y. Rb1/Rbl1/Vhl loss induces mouse subretinal angiomatous proliferation and hemangioblastoma. JCI Insight (2019) 22:e127889. doi: 10.1172/jci.insight.127889 PMC6948866 31613797

[65] Xie C Lu H Nomura A Hanse EA Forster CL Parker JB. Co-Deleting pten with Rb in retinal progenitor cells in mice results in fully penetrant bilateral retinoblastomas. Mol Cancer (2015) 14:93. doi: 10.1186/s12943-015-0360-y 25907958 PMC4411757

[66] Simeonova I Lejour V Bardot B Bouarich-Bourimi R Morin A Fang M. Fuzzy tandem repeats containing p53 response elements may define species-specific p53 target genes. PloS Genet (2012) 6:e1002731. doi: 10.1371/journal.pgen.1002731 PMC3386156 22761580

[67] Chen S Chen X Luo Q Liu X Wang X Cui Z. Retinoblastoma cell-derived exosomes promote angiogenesis of human vesicle endothelial cells through microRNA-92a-3p. Cell Death Dis (2021) 7:695. doi: 10.1038/s41419-021-03986-0 PMC8277798 34257272

[68] Shields CL Shields JA. Retinoblastoma management: advances in enucleation, intravenous chemoreduction, and intra-arterial chemotherapy. Curr Opin Ophthalmol (2010) 3:203–12. doi: 10.1097/ICU.0b013e328338676a 20224400

[69] Yu Y Wang Z Zheng Q Li J. GREB1L overexpression correlates with prognosis and immune cell infiltration in lung adenocarcinoma. Sci Rep (2021) 1:13281. doi: 10.1038/s41598-021-92695-x PMC8225624 34168239

[70] Kim C Xi L Cultraro CM Wei F Jones G Cheng J. Longitudinal circulating tumor DNA analysis in blood and saliva for prediction of response to osimertinib and disease progression in EGFR-mutant lung adenocarcinoma. Cancers (Basel) (2021) 13:3342. doi: 10.3390/cancers13133342 34283064 PMC8268167

[71] Gong WJ Liu JY Yin JY Cui JJ Xiao D Zhuo W. Resistin facilitates metastasis of lung adenocarcinoma through the TLR4/Src/EGFR/PI3K/NF-κB pathway. Cancer Sci (2018) 8:2391–400. doi: 10.1111/cas.13704 PMC6113506 29927028

[72] Kim C Giaccone G. Precision oncology in non-small-cell lung cancer: opportunities and challenges. Nat Rev Clin Oncol (2018) 6:348–9. doi: 10.1038/s41571-018-0008-0 29599475

[73] Stahel RA. Adenocarcinoma, a molecular perspective. Ann Oncol (2007) 18:ix147–9. doi: 10.1093/annonc/mdm310 17631568

[74] Zong S Jiao Y Liu X Mu W Yuan X Qu Y. FKBP4 integrates FKBP4/Hsp90/IKK with FKBP4/Hsp70/RelA complex to promote lung adenocarcinoma progression *via* IKK/NF-κB signaling. Cell Death Dis (2021) 6:602. doi: 10.1038/s41419-021-03857-8 PMC8192522 34112753

[75] Bouzidi A Magnifico MC Paiardini A Macone A Boumis G Giardina G. Cytosolic serine hydroxymethyltransferase controls lung adenocarcinoma cells migratory ability by modulating AMP kinase activity. Cell Death Dis (2020) 11:1012. doi: 10.1038/s41419-020-03215-0 33243973 PMC7691363

[76] Cheung CHY Juan HF. Quantitative proteomics in lung cancer. J BioMed Sci (2017) 1:37. doi: 10.1186/s12929-017-0343-y PMC5470322 28615068

[77] Yin J Lang T Cun D Zheng Z Huang Y Yin Q. pH-sensitive nano-complexes overcome drug resistance and inhibit metastasis of breast cancer by silencing akt expression. Theranostics (2017) 17:4204–16. doi: 10.7150/thno.21516 PMC5695007 29158820

[78] Gomes LR Rocha CRR Martins DJ Fiore APZP Kinker GS Bruni-Cardoso A. ATR mediates cisplatin resistance in 3D-cultured breast cancer cells *via* translesion DNA synthesis modulation. Cell Death Dis (2019) 6:459. doi: 10.1038/s41419-019-1689-8 PMC6561919 31189884

[79] Early Breast Cancer Trialists' Collaborative Group (EBCTCG). Effects of chemotherapy and hormonal therapy for early breast cancer on recurrence and 15-year survival: an overview of the randomised trials. Lancet (Lond Engl) (2005) 9472:1687–717. doi: 10.1016/S0140-6736(05)66544-0 15894097

[80] Gerber B Freund M Reimer T. Recurrent breast cancer: treatment strategies for maintaining and prolonging good quality of life. Dtsch Arztebl Int (2010) 6:85–91. doi: 10.3238/arztebl.2010.0085 PMC2832109 20204119

[81] Kast K Rhiem K. Familial breast cancer - targeted therapy in secondary and tertiary prevention. Breast Care (Basel Switzerland) (2015) 1:27–31. doi: 10.1159/000380756 PMC4395821 25960722

[82] Cardoso F Harbeck N Fallowfield L Kyriakides S Senkus E. Locally recurrent or metastatic breast cancer: ESMO clinical practice guidelines for diagnosis, treatment and follow-up. Ann Oncol (2012) 23:vii11–9. doi: 10.1093/annonc/mds232 22997442

[83] Qian D Zheng Q Wu D Ye B Qian Y Zhou T. Integrated analysis of ceRNA network reveals prognostic and metastasis associated biomarkers in breast cancer. Front Oncol (2021) 11:670138. doi: 10.3389/fonc.2021.670138 34055638 PMC8158160

[84] Groza IM Braicu C Jurj A Zanoaga O Lajos R Chiroi P. Cancer-associated stemness and epithelial-to-Mesenchymal transition signatures related to breast invasive carcinoma prognostic. Cancers (Basel) (2020) 10:3053. doi: 10.3390/cancers12103053 PMC7589570 33092068

[85] Zha W Li X Tie X Xing Y Li H Gao F. The molecular mechanisms of the long noncoding RNA SBF2-AS1 in regulating the proliferation of oesophageal squamous cell carcinoma. Sci Rep (2021) 1:805. doi: 10.1038/s41598-020-80817-w PMC7804443 33436941

[86] Vrána D Matzenauer M Neoral Č Aujeský R Vrba R Melichar B. From tumor immunology to immunotherapy in gastric and esophageal cancer. Int J Mol Sci (2018) 1:13. doi: 10.3390/ijms20010013 PMC6337592 30577521

[87] Siegel RL Miller KD Jemal A. Cancer statistics, 2019. CA Cancer J Clin (2019) 1:7–34. doi: 10.3322/caac.21551 30620402

[88] Chen JL Lin ZX Qin YS She YQ Chen Y Chen C. Overexpression of long noncoding RNA LINC01419 in esophageal squamous cell carcinoma and its relation to the sensitivity to 5-fluorouracil by mediating GSTP1 methylation. Ther Adv Med Oncol (2019) 11:1758835919838958. doi: 10.1177/1758835919838958 31019568 PMC6463338

[89] Xue Y Zhou X Xue L Zhou R Luo J. The role of pretreatment prognostic nutritional index in esophageal cancer: A meta-analysis. J Cell Physiol (2019) 11:19655–62. doi: 10.1002/jcp.28565 PMC6766897 31344989

[90] Yang SS Guo JG Liu JN Liu ZQ Chen EN Chen CY. Effect of induction chemotherapy in nasopharyngeal carcinoma: An updated meta-analysis. Front Oncol (2020) 591205. doi: 10.3389/fonc.2020.591205 33489889 PMC7820771

[91] Zhao Y Zhou Q Li N Shen L Li Z. Paranasal sinus invasion should be classified as T4 disease in advanced nasopharyngeal carcinoma patients receiving radiotherapy. Front Oncol (2020) 10:01465. doi: 10.3389/fonc.2020.01465 33240800 PMC7677568

[92] Li Z Zhou Z Wu X Zhou Q Liao C Liu Y. LMP1 promotes nasopharyngeal carcinoma metastasis through NTRK2-mediated anoikis resistance. Am J Cancer Res (2020) 7:2083–99. PMC7407352 32775002

[93] Li HP Huang CY Lui KW. Combination of epithelial growth factor receptor blockers and CDK4/6 inhibitor for nasopharyngeal carcinoma treatment. Cancers (Basel) (2021) 12:2954. doi: 10.3390/cancers13122954 PMC8231497 34204797

[94] Yu MC Yuan JM. Epidemiology of nasopharyngeal carcinoma. Semin Cancer Biol (2002) 6:421–9. doi: 10.1016/S1044579X02000858 12450728

[95] Wang X Liang H Xu W Ma X. Wallenda-nmo axis regulates growth *via* hippo signaling. Front Cell Dev Biol (2021) 9:658288. doi: 10.3389/fcell.2021.658288 33937258 PMC8085559

[96] Zhao F Feng Y Zhang X Liu X Li A. Kinesin superfamily member 18B (KIF18B) promotes cell proliferation in colon adenocarcinoma. Cancer Manag Res (2020) 12:12769–78. doi: 10.2147/CMAR.S261894 PMC7737937 33335427

[97] Collins K Jacks T Pavletich NP. The cell cycle and cancer. Proc Natl Acad Sci U S A (1997) 7:2776–8. doi: 10.1073/pnas.94.7.2776 PMC34145 9096291

[98] Kar S. Unraveling cell-cycle dynamics in cancer. Cell Syst (2016) 1:8–10. doi: 10.1016/j.cels.2016.01.007 27136683

[99] Wan L Pantel K Kang Y. Tumor metastasis: moving new biological insights into the clinic. Nat Med (2013) 11:1450–64. doi: 10.1038/nm.3391 24202397

[100] Mitra P Kalailingam P Tan HB Thanabalu T. Overexpression of GRB2 enhances epithelial to mesenchymal transition of A549 cells by upregulating SNAIL expression. Cells (2018) 8:97. doi: 10.3390/cells7080097 PMC6116178 30087284

[101] Giubellino A Burke TR Jr. Bottaro DP. Grb2 signaling in cell motility and cancer. Expert Opin Ther Targets (2008) 8:1021–33. doi: 10.1517/14728222.12.8.1021 PMC2764957 18620523

[102] Steeg PS. Targeting metastasis. Nat Rev Cancer (2016) 4:201–18. doi: 10.1038/nrc.2016.25 PMC7055530 27009393

[103] Massagué J Obenauf AC. Metastatic colonization by circulating tumour cells. Nature (2016) 7586:298–306. doi: 10.1038/nature17038 PMC5029466 26791720

[104] Zhang Z Li J Ou Y Yang G Deng K Wang Q. CDK4/6 inhibition blocks cancer metastasis through a USP51-ZEB1-dependent deubiquitination mechanism. Signal Transduct Target Ther (2020) 1:25. doi: 10.1038/s41392-020-0118-x PMC7064488 32296027

[105] Turajlic S Swanton C. Metastasis as an evolutionary process. Science (New York N Y) (2016) 6282:169–75. doi: 10.1126/science.aaf2784 27124450

[106] Nan X Wang J Liu HN Wong STC Zhao H. Epithelial-mesenchymal plasticity in organotropism metastasis and tumor immune escape. J Clin Med (2019) 5:747. doi: 10.3390/jcm8050747 PMC6571585 31130637

[107] Popeda M Stokowy T Bednarz-Knoll N Jurek A Niemira M Bielska A. NF-kappa b signaling-related signatures are connected with the mesenchymal phenotype of circulating tumor cells in non-metastatic breast cancer. Cancers (Basel) (2019) 12:1961. doi: 10.3390/cancers11121961 PMC6966426 31817685

[108] Yao H Han B Zhang Y Shen L Huang R. Non-coding RNAs and autophagy. Adv Exp Med Biol (2019) 1206:199–220. doi: 10.1007/978-981-15-0602-4_10 31776987

[109] Onorati AV Dyczynski M Ojha R Amaravadi RK. Targeting autophagy in cancer. Cancer (2018) 16:3307–18. doi: 10.1002/cncr.31335 PMC6108917 29671878

[110] Kim KH Lee MS. Autophagy–a key player in cellular and body metabolism. Nat Rev Endocrinol (2014) 6:322–37. doi: 10.1038/nrendo.2014.35 24663220

[111] Parzych KR Klionsky DJ. An overview of autophagy: morphology, mechanism, and regulation. Antioxid Redox Signal (2014) 3:460–73. doi: 10.1089/ars.2013.5371 PMC3894687 23725295

[112] Morris BJ. Human renin protein and gene structures: present and future targets for renin blockade in treatment of hypertension. J Hypertens Suppl (1989) 2:S9–14. doi: 10.1097/00004872-198904002-00003 2666620

[113] Frankel LB Lubas M Lund AH. Emerging connections between RNA and autophagy. Autophagy (2017) 1:3–23. doi: 10.1080/15548627.2016.1222992 PMC5240835 27715443

[114] Zhang J Wang P Wan L Xu S Pang D. The emergence of noncoding RNAs as heracles in autophagy. Autophagy (2017) 6:1004–24. doi: 10.1080/15548627.2017.1312041 PMC5486373 28441084

[115] Yang L Wang H Shen Q Feng L Jin H. Long non-coding RNAs involved in autophagy regulation. Cell Death Dis (2017) 10:e3073. doi: 10.1038/cddis.2017.464 PMC5680586 28981093

[116] Zou Z Yuan Z Zhang Q Long Z Chen J Tang Z. Aurora kinase a inhibition-induced autophagy triggers drug resistance in breast cancer cells. Autophagy (2012) 12:1798–810. doi: 10.4161/auto.22110 PMC3541289 23026799

[117] Wang K Ma L Tang J Yu Q Shen Y Wei Y. LncRNA00518 promotes cell proliferation through regulating miR-101 in bladder cancer. J Cancer (2020) 6:1468–77. doi: 10.7150/jca.35710 PMC6995372 32047553

[118] Vitamin e supplementation of premature infants. Nutr Rev (1988) 3:122–3. doi: 10.111/j.1753-4887.1988.tb05397.x 3386904

[119] Yin H Wang X Zhang X Wang Y Zeng Y Xiong Y. Integrated analysis of long noncoding RNA associated-competing endogenous RNA as prognostic biomarkers in clear cell renal carcinoma. Cancer Sci (2018) 10:3336–49. doi: 10.1111/cas.13778 PMC6172067 30152187

[120] Wang QF Wang QL Cao MB. LncRNA PITPNA-AS1 as a potential diagnostic marker and therapeutic target promotes hepatocellular carcinoma progression via modulating miR-448/ROCK1 axis. Front Med (Lausanne) (2021) 8:668787. doi: 10.3389/fmed.2021.668787 34055841 PMC8149744

[121] Simion V Zhou H Haemmig S Pierce JB Mendes S Tesmenitsky Y. A macrophage-specific lncRNA regulates apoptosis and atherosclerosis by tethering HuR in the nucleus. Nat Commun (2020) 1:6135. doi: 10.1038/s41467-020-19664-2 PMC7708640 33262333

[122] Fichtner AS Karunakaran MM Gu S Boughter CT Borowska MT Starick L. Alpaca (Vicugna pacos), the first nonprimate species with a phosphoantigen-reactive Vγ9Vδ2 T cell subset. Proc Natl Acad Sci U S A (2020) 12:6697–707. doi: 10.1073/pnas.1909474117 PMC7104304 32139608

[123] Yao ZT Yang YM Sun MM He Y Liao L Chen KS. New insights into the interplay between long non-coding RNAs and RNA-binding proteins in cancer. Cancer Commun (Lond Engl) (2022) 2:117–40. doi: 10.1002/cac2.12254 PMC8822594 35019235

[124] Toden S Goel A. Non-coding RNAs as liquid biopsy biomarkers in cancer. Br J Cancer (2022) 3:351–60. doi: 10.1038/s41416-021-01672-8 PMC8810986 35013579

